# Effect of Ultrasound Combinated with Sodium Hypochlorite Treatment on Microbial Inhibition and Quality of Fresh-Cut Cucumber

**DOI:** 10.3390/foods12040754

**Published:** 2023-02-09

**Authors:** Chunhong Zhang, Wanfu Hou, Wenting Zhao, Shuang Zhao, Pan Wang, Xiaoyan Zhao, Dan Wang

**Affiliations:** 1 College of Food Science, Shenyang Agricultural University, Shenyang 110866, China; 2 Beijing Key Laboratory of Fruits and Vegetables Preservation and Processing, Key Laboratory of Vegetable Postharvest Processing, Ministry of Agriculture and Rural Affairs, Institute of Agri-food Processing and Nutrition, Beijing Academy of Agriculture and Forestry Sciences, Beijing 100097, China

**Keywords:** fresh-cut cucumber, microbial changes, quality

## Abstract

The influence of ultrasound combined with sodium hypochlorite (US-NaClO) treatment on microorganisms and quality of fresh-cut cucumber during storage were investigated. Ultrasound (400 W, 40 kHz, US: 5, 10 and 15 min) and sodium hypochlorite (NaClO: 50, 75, 100 ppm) were used to treat fresh-cut cucumber in a single or combined treatment and stored at 4 °C for 8 days and analyzed for texture, color and flavor. The results showed that US-NaClO treatment had a synergistic effect on the inhibition of microorganisms during storage. It could significantly reduce (*p* < 0.05) the number of microorganisms by 1.73 to 2.17 log CFU/g. In addition, US-NaClO treatment reduced the accumulation of malondialdehyde (MDA) during storage (4.42 nmol/g) and water mobility, and maintained the integrity of the cell membrane, delayed the increase of weight loss (3.21%), reduced water loss, thus slowing down the decline of firmness (9.20%) of fresh-cut cucumber during storage. The degradation of chlorophyll (6.41%) was reduced to maintain the color of freshly cut cucumbers. At the same time, US-NaClO could maintain the content of aldehydes, the main aromatic substance of cucumber, and reduced the content of alcohols and ketones during storage. Combined with the electronic nose results, it could maintain the cucumber flavor at the end of the storage period and reduce the odor produced by microorganisms. Overall, US-NaClO was helpful to inhibit the growth of microorganisms during storage, improve the quality of fresh-cut cucumber.

## 1. Introduction

In recent years, fresh-cut vegetables have met consumers’ demands for health and environmental protection due to their nutritional, convenient and fresh features, and thus their sales have increased significantly [1]. Fresh-cut cucumber has high water content, is rich in protein, propanoldiacid, vitamins, minerals and other nutrients, and is widely used in Chinese dishes and Western salads [2,3]. However, fresh-cut cucumber is still subject to tissue respiration and transpiration after processing, which aggravates tissue damage, discoloration and nutrient loss during processing. Moreover, it increases the risk of microbial contamination because of cutting processing, which easily causes tissue softening, deterioration and odor, reduces the quality of fresh-cut cucumber during storage and shortens the shelf life [4,5].

Fresh-cut products need to be disinfected before sale to ensure food safety [6,7]. A variety of disinfection technologies for fresh-cut products have been studied, such as high-pressure treatment, ozone, ultraviolet-C radiation, etc. However, they are not widely used in factories because of high cost or possible negative impact on products or consumer health. For example, although ozone is widely used in the disinfection of fruits and vegetables, its stability is low and it is not easy to store. At the same time, due to its strong oxidation, it may cause certain damage to the product quality [8]. Chlorine is widely used in the disinfection of fresh-cut fruits and vegetables because of low price and convenience. It produces small neutral molecular hypochlorite when dissolved in water, which is more effective in the sterilization of microorganisms [9,10]. However, because there are pores and folds on the surface of fruits and vegetables, which can wrap bacteria and make it difficult for NaClO solution to enter, there are some limitations on the bactericidal effect [11]. Meanwhile, long-term use of NaClO with a higher concentration will produce a peculiar smell and threaten the product quality [12]. It reacts with natural organic substances (NOM) in water to produce some toxic by-products, such as trihalomethane, trichloromethane and other carcinogenic by-products [13]. Therefore, the current research focus is to use low concentration chlorine to disinfect fresh-cut products, reduce processing costs and improve product safety [14].

As a new non-thermal technology, US has the unique advantages of low cost, environmental protection and non-toxic. It is widely used in food processing and plays a good role in maintaining the color, flavor, nutrition and microbial inactivation of fruits and vegetables [15,16,17]. It can produce a cavitation effect in 20–100 KHz, and produce a local high temperature and pressure in the liquid, thus leading to the destruction of some microbial cell wall structures and inactivation or death of bacteria [18,19]. However, the disinfection effect of US on the surface of fruits and vegetables has certain limitations, so it is often combined with other technologies to improve sterilization. The results showed that the decontamination effect of US combined with essential oil on *Escherichia coli* on tomato was much better than that of US alone [20]. Some studies have reported that US combined with peracetic acid [21] or a modified atmosphere [22] and NaClO combined with a modified atmosphere can disinfect fresh-cut cucumber and cilantro [23] and maintain their quality. In addition, there have been studies on the use of US-NaClO to inhibit bacteria and maintain the quality of yellow melon, winter jujube, lettuce, etc. However, the effect of US-NaClO on microbial inhibition and the quality of fresh-cut cucumber has not been reported.

In this study, the inhibitory effect of ultrasonic combined with low concentration of sodium hypochlorite on microorganisms and quality change of fresh-cut cucumber during storage period were analyzed. The results would provide useful information for industrial production.

## 2. Materials and Methods

### 2.1. Raw Materials

The cucumber (*Cucumis sativus* L.) variety “Jingyan Xiamei No. 2”, Mature cucumbers (36–38 cm) was purchased from the local supermarket (Guoxiangsiyi, Beijing, China). It was planted at a farm in Changping District, Beijing from the end of February to the end of March (the growth period was about 120 days). It was cut and picked at about 0.5 cm from the handle and stored in a refrigerator at 4 °C before the experiment. Cucumbers were washed with deionized water, and then rinsed repeatedly with sterile water for three times. They were allowed to dry in a biosafety cabinet. The cucumbers were sliced into even slices (3–5 mm) with a sterile scalpel, and then used as fresh-cut cucumbers.

### 2.2. Sample Treatment

The processing of the sample was based on the method of Fan et al. [22] and slightly modified. The cucumber samples were divided into four treatment groups, and the sampling process was completely random and repeated three times. Group A was with an ultrasonic device (KQ-500DE, Kunshan Ultrasonic In substance Ltd., Jiangsu, China), the 150 g sample was completely immersed in a beaker containing 600 mL of sterile distilled water and placed together in an ultrasonic bath (42 × 31.5 × 14 cm) containing 20 L of distilled water. The working frequency was set at 40 kHz, power at 400 W (Ultrasonic density 0.5 W/cm^2^, ultrasonic intensity 50 W/cm^2^, amplitude 20 μm) and a water bath treatment was conducted for 5, 10 and 15 min (marked as US 5 min, US 10 min, US 15 min). Group B was immersed in NaClO solution with a concentration of 50, 75, 100 ppm (to avoid affecting the experimental effect, the reagents were prepared on the day of the experiment) for 5 min (marked as NaClO-50, 75, 100 ppm), Group C was the best time for screening US treatment (40 kHz, 400 W, the best time of US was determined by preliminary experimental screening) and combined with 50, 75, 100 ppm NaClO solution, respectively, while in group D, untreated samples were used as the control group. After treatment, all samples were dried in the biosafety cabinet (25 °C for 30 min) and put into polyethylene plastic bags for storage at 4 °C for 8 days for subsequent analysis. The quality parameters of fresh-cut cucumber samples were evaluated at 0, 4 and 8 days, and equal portions of the samples were frozen at −80 °C in liquid nitrogen for subsequent nutrient index analysis.

### 2.3. Quantification of Microorganisms

The total number of colonies in fresh-cut cucumber samples was measured. Then, 15 g of sample was placed into a sterile homogeneous bag (S05D, Land Bridge Technology Co., Ltd., Beijing, China). Further, 135 mL of sodium chloride solution (0.8%) was added into it [24,25], using a beating homogenize (BagMixer 400 W, Interscience Lab Inc., Hanover, MA, USA) for 5 min to mix. Next, 1 mL homogenate solution was diluted with 9 mL NaCl solution at a ratio of 1:10, then 0.05 mL diluted suspension was spread on nutrient agar and incubated at 37 °C for 48 h.

### 2.4. Evaluation of Quality Parameters

#### 2.4.1. Weight Loss and Firmness

The loss of the sample weight was determined by the method of Wang et al. [26], and the result is expressed in % by weighing it every 4 days.

The firmness of the fresh-cut cucumber was determined by a texture analyzer (Stable Micro Systems Ltd., Godalming, UK) [27]. Using a P/2 probe for measurement, based on previous multiple tests, the parameters were as follows: pre-test speed 10 mm/s, test speed 2 mm/s; post-test speed 10 mm/s. Then, three samples were randomly selected from the treatment group for testing, and it was ensured that each puncture was close to each other. The maximum force at the puncture depth of 3 mm was taken as the firmness and expressed as N.

#### 2.4.2. Malondialdehyde Content

The content of malondialdehyde (MDA) was determined by using a kit (Solaibao Technology Co. Ltd., Beijing, China) [28,29]. Further, 0.5 g of tissue was weighed, and 1 mL of extract was added for ice bath homogenization, and then centrifuged at 8000× *g* for 10 min at 4 °C. The supernatant was taken, the MDA working solution was added into a 100 °C water bath for 60 min, and after cooling, the liquid was placed in an ice bath and centrifuged at 10,000× *g* for 10 min at room temperature. Absorbance values were measured at 532 and 600 nm using a UV-1800 spectrophotometer (Shimadzu, Kyoto, Japan).

#### 2.4.3. Analysis of Water Mobility and Distribution

The water status and distribution of fresh-cut cucumber samples during storage were determined by low-field nuclear magnetic resonance (LFNMR) and magnetic resonance imaging (MRI) [30]. A LF-NMR analyzer (NMI20-025V-I, Niumag Co., Ltd., Suzhou, China) was used to measure the transverse relaxation time (T_2_) of fresh-cut cucumber samples. The samples (6 g ± 0.05 g) were placed in the glass tube, and then placed in the magnet chamber (32 °C). The Carre–Purcelle–Meiboome–Gill (CPMG) sequence was used for determination. The transverse proton hydrogen density images of fresh-cut cucumber samples from different treatment groups were analyzed by MRI to observe the water distribution of different samples.

#### 2.4.4. Color Evaluation

The CM-3700d desktop colorimeter (Konica Minolta Sensing, Inc., Osaka, Japan) was used to measure the color of fresh-cut cucumber samples in different treatment groups every 4 days [31]. Before the measurement, a white standard was used to calibrate, and L*: brightness, a*: red-green color, b*: yellow-blue color to represent the color of the sample. Total color change (ΔE) was calculated as follows. Each sample has 12 points, and 6 samples were measured. The average value was used as the color value.

(1) $$∆E=\sqrt{{\left(L_{0}^{*}-L^{*}\right)}^{2}+{\left(a_{0}^{*}-a^{*}\right)}^{2}+{\left(b_{0}^{*}-b^{*}\right)}^{2}}$$

where $L_{0}^{*}$, $a_{0}^{*}$ and $b_{0}^{*}$ were color values of fresh-cut cucumber samples on 0 d.

#### 2.4.5. Chlorophyll Concentration

The chlorophyll kit (Solaibao Technology Co. Ltd., Beijing, China) was used to determine the chlorophyll content in fresh-cut cucumber samples. The frozen tissue stored at −80 °C was put into the extract, ground fully under dark or low light conditions and then extracted for 3 h. When the tissue residue was close to white, it indicated that the extraction was complete. The chlorophyll content was determined at 645 and 663 nm using the UV-1800 spectrophotometer (Shimadzu, Kyoto, Japan).

#### 2.4.6. Flavor Analysis

The flavor information of fresh-cut cucumber samples in different treatment groups was analyzed by the electronic nose system, which consisted of 10 sensors [24], 6 g of fresh-cut cucumber was weighed, diced and put it into a 50 mL headspace bottle, and it was allowed to stand at room temperature for 30 min to equilibrate the volatile components in the sample. In this study, the parameters of the electronic nose were set as follows: measured at room temperature 25 °C, the cleaning time of the sensor was 120 s, the automatic zero setting was 5 s, the time of the sample to be measured was 5 s, the flow rate of the carrier gas was 400 mL/min during the measurement and the detection was 180 s. The measurement was repeated for three times for each group of samples, and data of 138~140 s were selected for analysis.

#### 2.4.7. Volatile Organic Compounds

The method of Wang et al. [2] was referred to for the determination of volatile organic compounds, a gas chromatography-ion mobility spectrometry (GC-IMS) equipped with mxt-5 (15 m × 0.53 mm × 1 μm) (FlavourSpec^®^, G.A.S., Dortmund, Germany) was used to analyze the flavor changes of fresh-cut cucumber samples during storage under different treatments.

The frozen fresh-cut cucumber samples were thawed at room temperature and ground into slurry, and then 2.0 mL of cucumber slurry was taken and put into a 20 mL headspace bottle. Then, the injection conditions of all samples were set as follows: incubation temperature 40 °C, oscillator speed 500 r/min, incubation time 20 min. After incubation, 1 mL of headspace gas was automatically injected through a syringe at 85 °C, no split flow mode. The chromatographic column temperature was maintained at 60 °C, the carrier gas was high pure nitrogen (99.99%), and the carrier gas flow was set as: 0–2 min 2 mL/min; 2–10 min 10 mL/min; 10–20 min 100 mL/min; 20–25 min 150 mL/min.

The retention index (RI) was calculated with the n-Ketones C4-C9 (Ruisi Taikang Technology Co., Ltd. Beijing, China), and the volatile organic compounds were identified based on RI, drift time (Dts) and GC-IMS library.

#### 2.4.8. Sensory Analysis

A panel of 10 reviewers with long-term experience in sensory evaluation conducted a sensory evaluation on fresh-cut cucumber samples from different treatment groups, including appearance, color, water loss (texture), smell [5]. The full score of each item was 5 points, and the total cumulative score was 20 points. The evaluation grade is: 1 point, unqualified, the sample is rotten, the fresh cucumber color is lost, the water is lost seriously and there is an unacceptable rotten smell. Two points, poor, water stains appear on the sample, the color is dark and yellow, the surface and edge are wilted and there is an odor. Three points, medium, slightly watered surface, slightly yellow color, soft surface, slightly peculiar smell. Four points, good, slightly dehydrated, bright green in color and the smell of the cucumber is not obvious. Five points, excellent, full in texture, green in color, with obvious smell of fresh cucumber.

### 2.5. Statistical Analysis

All data were analyzed with Origin 2018 software (Originalab, Northampton, MA, USA), and expressed as means ± standard deviation (*n* = 3), SPSS 26.0 (IBM Corp., Armonk, NY, USA), for significance analysis (Duncan’s test, *p* < 0.05).

## 3. Results and Discussion

### 3.1. Microbial Analysis

**Figure 1:**
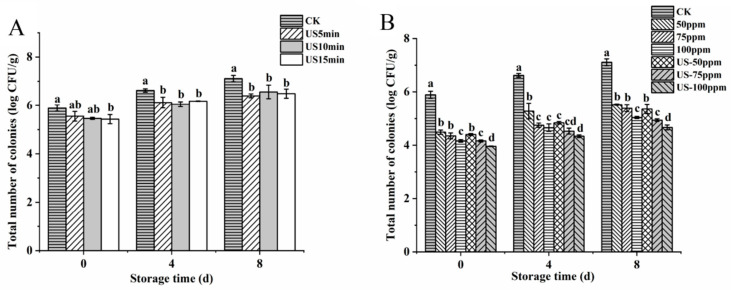
Effects of US treatment (**A**); NaClO and US-NaClO treatment (**B**) on total number of colonies of fresh-cut cucumber during storage. Different letters indicate significant differences among treatments at same storage time (*p* < 0.05).

The effect of US treatment on the total bacterial count of fresh-cut cucumber is shown in Figure 1A. At the initial stage of storage, the total bacterial count of fresh-cut cucumber after US treatment was lower than that of the control, with a maximum difference of 0.45 log CFU/g. This phenomenon might be attributed to the cavitation effect of US. US treatment would produce cavitation bubbles in the liquid medium, which would lead to extremely high pressure and temperature in some areas, leading to the destruction of the microbial structure, increasing the permeability of the cell membrane and inactivating microorganisms [32]. Cao et al. [33] also showed similar results; they found that US treatment could effectively reduce the number of microorganisms in strawberry fruit and delay its decay. The total bacterial count of all samples exhibited an increasing trend with prolonging storage time. At the end of the 8-day storage period, the total bacterial count of the control, US 5 min, US 10 min and US 15 min were 7.11, 6.40, 6.56 and 6.49 log CFU/g, respectively, indicating that the total bacterial count of the samples treated with US was significantly lower than that of the control (*p* < 0.05). US 5 min, US 10 min and US 15 min treatments had no significant difference in the reduction of total bacterial count during storage (*p* > 0.05), so US 5 min was used as the treatment condition for subsequent experiments.

As shown in Figure 1B, compared with the control group, both NaClO (50–100 ppm) immersion for 5 min and US-NaClO (US 5 min-50, 75, 100 ppm) treatments significantly (*p* < 0.05) reduced the total bacterial count in the initial stage of fresh-cut cucumber by 1.40, 1.53, 1.72, 1.49, 1.73 and 1.93 log CFU/g, respectively. The results showed that the combined treatment has a certain synergistic effect compared with the single NaClO treatment. It might be due to the cavitation effect produced by US treatment, where separating cells from the sample surface would change the permeability of the cell membrane and expose the contents of cells in the disinfectant, thus improving the bacteriostatic effect [34]. Compared with the control group, on the 8th day, the total bacterial count of NaClO alone and combined treatment decreased by 1.59, 1.72, 2.07, 1.75, 2.17, 2.44 log CFU/g, respectively. Similar to the results of Rosario et al. [35], they found that the US-NaClO treatment can significantly reduce the total bacterial count of yellow melon while maintaining its quality. Furthermore, according to the results, US combined with 75 ppm NaClO could achieve the bacteriostatic effect of 100 ppm NaClO. Therefore, US-75 ppm and 100 ppm NaClO were selected for the quality test.

### 3.2. Texture Properties Analysis

#### 3.2.1. Firmness

Table 1 shows the changes of weight loss and firmness of fresh-cut cucumber. Firmness is an important parameter to measure food quality, reflecting the strength and thickness of the cell wall and the degree of adhesion between cells [36]. At the initial stage of storage, there was no significant difference in firmness between the control group and the treatment group (*p* > 0.05), but with the increase of storage time, the firmness of all samples showed a downward trend. The firmness of the control group decreased by 21.10% at the end of the storage period, while the firmness of US and NaClO alone or in combination decreased by only 13.76%, 12.45% and 9.20%, which was significantly lower than that of the control group (*p* < 0.05). The decrease in firmness of fresh-cut cucumber during storage may be due to the growth of microorganisms or the loss of water. At the same time, some studies have shown that US treatment (>10 min) would produce more cavitation effects, destroy the original stable structure of cells [37]. Rosario et al. [35] used US for 5 min combined with NaClO treatment to maintain the firmness of yellow melon; US has a protective effect on cell wall. It can inhibit the activity of the cell wall decomposing enzymes to maintain fruit and vegetable hardness, which was similar to the results of this study.

#### 3.2.2. Weight Loss

Fresh-cut fruits and vegetables are prone to weight loss during storage after mechanical cutting, which is due to the increase of cell transpiration and respiration, resulting in a large amount of water loss in the fruits and vegetables, thus increasing the weight loss rate [38], and some studies have shown that the loss of water would accelerate the softening of the pulp or the senescence of the fruit, leading to the reduction of fruit firmness [39,40]. Table 1 shows that the weight loss rate of both the control group and treatment group increased gradually with the increase of storage time. At the 8th day of storage, the weight loss of the control group and several treatment groups was 4.57%, 4.65%, 4.09%, 3.21%, respectively. The weight loss of fresh-cut cucumber after US-NaClO treatment was significantly lower (*p* < 0.05) than that of the control group. This might be due to the effective maintenance of hydrogen bonds between water molecules and macromolecules in fruits and vegetables after US treatment, as well as reducing water loss and maintaining firmness [41]. This was similar to the result shown by Wang et al. [26], who found that US combined with acetic acid and gibberellic acid could reduce the weight loss of green asparagus during storage.

#### 3.2.3. Malondialdehyde Content

**Figure 2:**
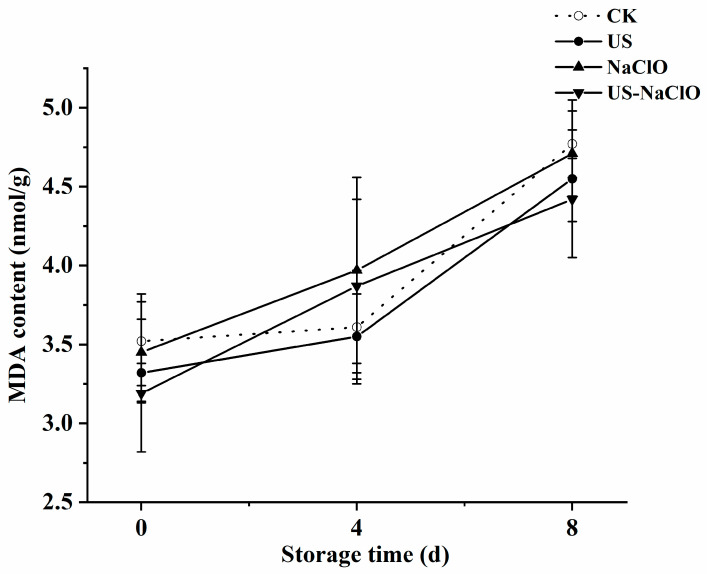
Effects of US, NaClO and US-NaClO treatment on MDA content of fresh-cut cucumber during storage.

Malondialdehyde (MDA) is the product of membrane lipid peroxidation, and a large amount of accumulation will have a negative impact on the membrane structure [42], Figure 2 shows that the content of MDA increased at the end of the storage period. After 8 days of storage, the content of MDA in the control group, US, NaClO treatment group and US-NaClO treatment group was 4.77, 4.55, 4.71 and 4.42 nmol/g, respectively, and US-NaClO treatment could reduce the accumulation of MDA. This might be because US treatment can maintain the integrity of cells and reduced the degree of membrane lipid oxidation of fresh-cut cucumber. At the same time, the integrity of the cell membrane was also related to changes in firmness, which was consistent with the previous firmness results [43].

#### 3.2.4. Water Mobility and Distribution

**Figure 3:**
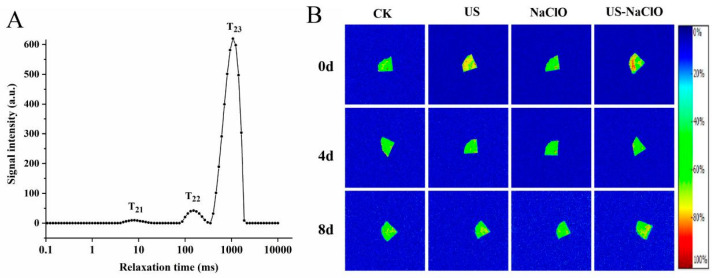
Distribution of transverse relaxation times (T_2_) in fresh-cut cucumber (T_21_: bound water, T_22_: fixed water, T_23_: free water) (**A**) and pseudo-color images of fresh-cut cucumber under different treatments during storage (**B**).

The relaxation time (T_2_) determines the water mobility and the degree of binding with its substrate. The changes between different tissues are observed through the mobility of water and the distribution of water between tissues to evaluate the maturity and the degree of damage and decay of fruits and vegetables. The role of monitoring the quality of fruits and vegetables. LF-NMR is a new detection technology with high efficiency, safety and rapidity, which can analyze the rule of water molecule migration through the change of relaxation time [44]. Figure 3A shows the distribution of transverse relaxation time (T_2_) of fresh-cut cucumber during 8-day storage measured by LF-NMR. The results showed that there were three proton relaxation peaks (T_21_, T_22_, T_23_) on the T_2_ distribution curve, which represented bound water (T_21_, 3 ms–25 ms), fixed water (T_22_, 70 m–300 ms) and free water (T_23_, 300 ms–2000 ms), respectively. A_21_, A_22_ and A_23_ represented the percentage of water under the three water conditions.

Table 2 exhibited the effects of different treatments on the relaxation time T_23_ and free water ratio A_23_ of fresh-cut cucumber during storage. During storage, T_23_ of the samples in the treatment group generally showed a trend of rising first and then declining, while A_23_ was significantly different from the control group (*p* < 0.05), which was due to the rupture of the cell membrane during storage and the increase of membrane permeability [22]. At the end of the 8-day storage period, there was no significant difference (*p* > 0.05) of T_23_ between US-NaClO treatment and the control group, while A_23_ of US-NaClO was significantly lower than that of the treatment group, indicating that US-NaClO improved membrane permeability and reduced the respiratory rate, effectively reducing the migration of bound water to free water, maintaining the stability of water and non-water components, thus reducing the proportion of free water, which was similar to the results of Feng et al. [41].

MRI can more clearly observe the change of water distribution in fresh-cut cucumber tissues during storage. Figure 3B shows the pseudo-color images of fresh-cut cucumber after different treatments. Different colors represent the change of water content. The redder the pseudo-color is, the higher the water proton density is. As shown in the figure, at the beginning of the storage period, the control group, US, US-NaClO showed a relatively high water proton density. Specifically, US-NaClO maintained high water proton density. However, at the end of the storage period, the proton density of water in several treatment groups decreased to varying degrees, indicating that water migration occurred during the storage period. These results showed that the US-NaClO treatment maintained higher water content and reduced a changein the water state.

### 3.3. Color Analysis

#### 3.3.1. Color

**Figure 4:**
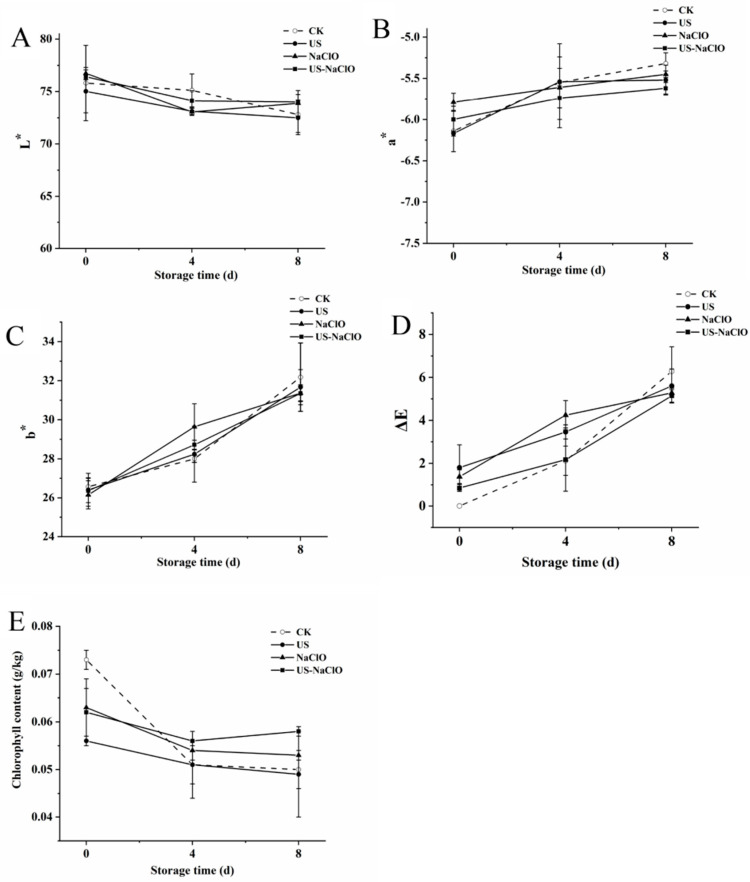
Effects of US, NaClO and US-NaClO treatments on L* (**A**), a* (**B**), b* (**C**), ΔE (**D**) and chlorophyll content (**E**) of fresh-cut cucumber during storage.

The color of fresh-cut fruits and vegetables is a key quality indicator to determine consumers’ visual acceptance [44]. As could be seen from Figure 4A–D, on day 0, the color difference of all treatment groups was not obvious, but at the end of the storage period, the fresh-cut cucumber samples of the control group and treatment group showed a trend of L* value decreasing, a* value, b* value and ΔE value increasing. At the end of the storage period, the value of a* in the control group and the treatment group increased by 13.35%, 10.53%, 5.87% and 6.33%; the decrease of the green color may be caused by the degradation of chlorophyll. The b* and ΔE values of the control group were higher than those of US-NaClO. The b* values of the two groups increased by 17.43% and 15.86%, respectively, and the ΔE values were 6.28 and 4.14, respectively, while the value of L* decreased slowly, it showed that US-NaClO treatment could slow down the color change of fresh-cut cucumber. Francisco et al. [45] reported that using US combined with NaClO treatment can maintain the color of fresh arugula.

#### 3.3.2. Chlorophyll Content

Chlorophyll is a green pigment and a key nutrient index in vegetables. The degradation of chlorophyll will reduce the appearance and quality of fresh-cut fruits and vegetables [4]. Figure 4E shows that at the initial stage, the chlorophyll content of the control group, US, NaClO treatment group and US-NaClO group was 0.073, 0.056, 0.063 and 0.062 mg/kg, respectively. However, with the increase of the storage period, the chlorophyll content of the samples in the control group and treatment group showed a downward trend, and the chlorophyll content of the control group decreased significantly on the 4th day. On the 8th day, the chlorophyll content of the control group and the treatment group lost 31.88%, 13.20%, 16.4% and 6.41%, respectively. This phenomenon might be due to the mechanical and chemical effects of US treatment, which can effectively inhibit or degrade chlorophyll-related enzymes, so as to maintain the content of chlorophyll during storage. At the same time, US-NaClO could also inhibit the content of ethylene [4,9,46]. Similar to the results of Alenyorege et al. [47], they used US combined with NaClO to maintain the chlorophyll degradation of Chinese cabbage during storage. Therefore, US-NaClO could effectively maintain the color protection of fresh-cut cucumber during storage, which had a positive impact.

### 3.4. Flavor Analysis

#### 3.4.1. Flavor

**Figure 5:**
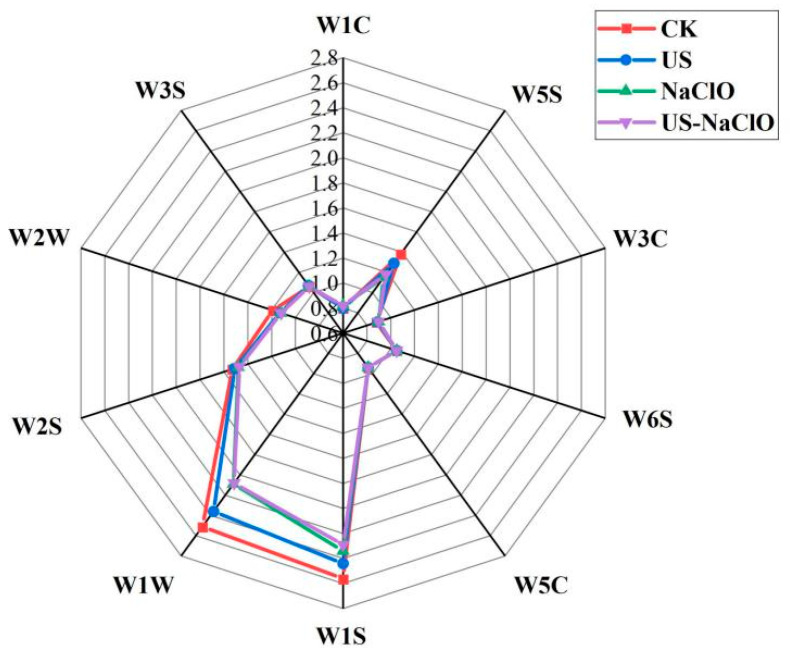
Electronic nose radar plot of the effect of US, NaClO and US-NaClO treatments on the flavor of fresh-cut cucumber on the 8th day of storage.

Volatile compounds are produced by secondary metabolites of fruits and vegetables [48]. The flavor changes of fresh-cut cucumber on the 8th day after different treatments are shown in Figure 5. It could be observed that the sensor response values of W1W, W1S and W5S were significantly higher than other sensors. W1W, W1S and W5S indicated that volatile substances, such as sulfide, terpene compounds, nitrogen oxides and methane, changed significantly after 8 days of storage, while the response values of W1W, W1S and W5S sensors in the US-NaClO treatment group were lower than those in the control group. It showed that the US-NaClO treatment group could reduce the volatilization of flavor substances of fresh-cut cucumber during storage, which might be that the combined treatment group could reduce the water mobility and inhibit the growth of microorganisms [9].

#### 3.4.2. Volatile Organic Compounds

**Figure 6:**
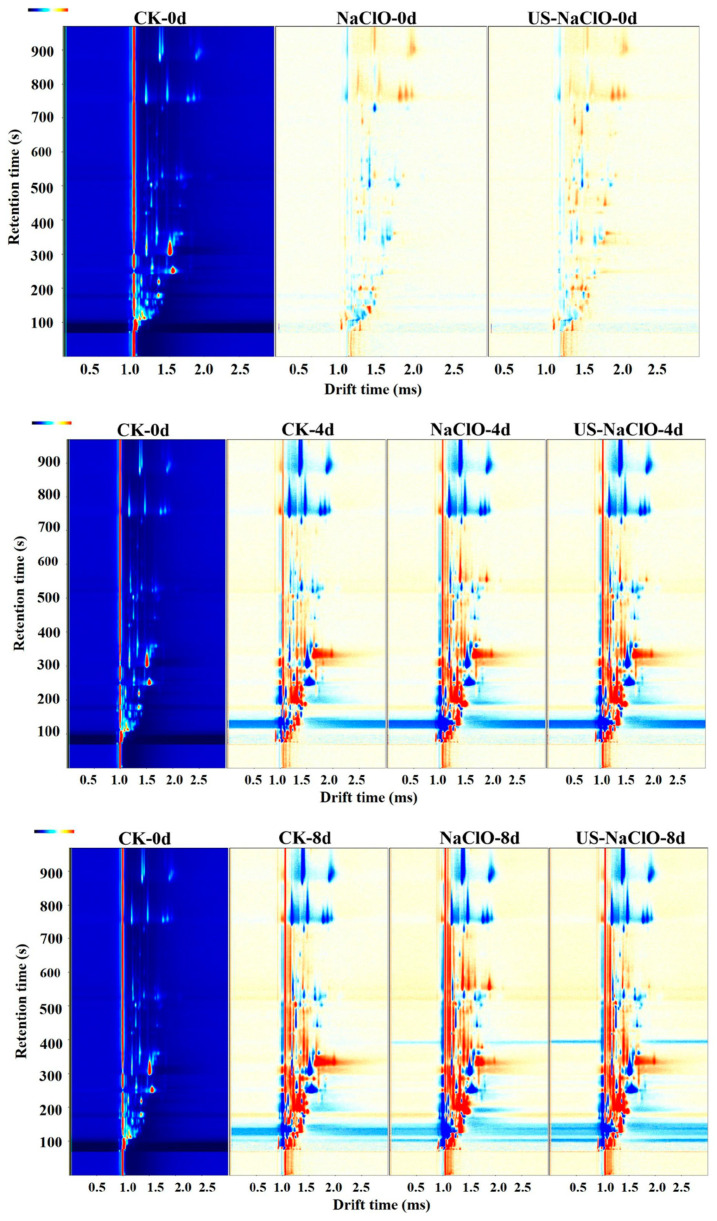
Comparison of the two-dimensional difference spectra of volatile organic compounds GC-IMS in fresh-cut cucumber under different treatments.

Volatile organic compounds (VOCs) are closely related to fresh-cut products and are the key factors influencing consumers’ acceptance of these products [24]. Three treatment groups (control group, NaClO treatment group and US-NaClO treatment group) with large flavor differences were screened by electronic nose results. The differences of VOCs of fresh-cut cucumber during storage were compared by GC-IMS. In order to more intuitively compare the differences of VOCs during storage, the difference comparison chart was used for analysis. Figure 6 shows the two-dimensional difference contrast diagram formed by subtracting the reference from the 0-day spectrogram of fresh-cut cucumber. When the background after cutting was white, it indicated that the concentration of this volatile substance was the same among treatment groups. When a red substance appeared, it indicated that the concentration of the substance was higher than the reference value, while blue indicated that the concentration of the substance was lower than the reference value, and the darker the color was, the greater the difference in the concentration of the substance [49]. When some substances appeared at different migration times, and form two or more signal peaks in the spectrum, forming a dimer or even a multimer, this was due to the fact that two substances with higher concentrations would share a proton or electron. It can be clearly seen from the figure that with the extension of the storage period, the VOCs of fresh-cut cucumber in the control group and between treatments were significantly different.

GC-IMS can qualitatively analyze VOCs in different samples, as shown in Table 3. A total of 51 substances were identified: 26 aldehydes, 10 alcohols, seven ketones, five esters, one furan, one pyrazine and one other compound, including some monomers and dimers.

**Figure 7:**
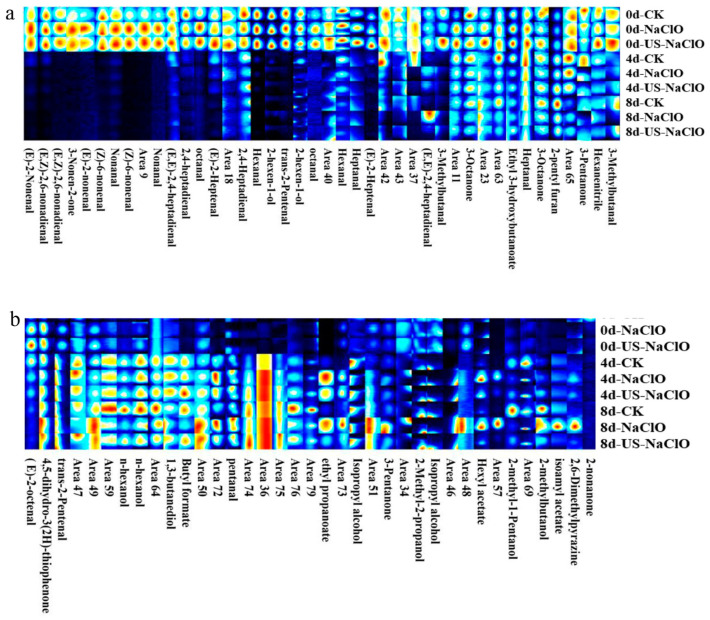
Fingerprints of volatile organic compounds in fresh-cut cucumbers under different treatments. (**a**,**b**): magnified regions.

Although the differences of VOCs in different treatment groups during storage could be directly observed in the two-dimensional difference map, it was difficult to make accurate judgments on some substances on the spectrum, and the use of the fingerprint could solve this problem very well. Therefore, the ion peak in the spectrum was selected to generate the fingerprint. Each row in Figure 7 represents all the signal peaks of the selected sample, and each column represents the signal peak intensity of the same volatile substance in fresh-cut cucumber samples in different treatment groups.

It could be seen from the figure that the VOCs of fresh-cut cucumber changed significantly with the extension of the storage period. The contents of Hexanal, trans-2-pentenal, 3-Pentanone, 3-Methylbutanal, Heptanal, 2-hexen-1-ol, (E)-2-Heptenal, 2-pentyl furan, 2,4-Heptadienal, 3-Octanone, (Z)-6-nonenal, (E,Z)-2,6-nonadienal, octanal, (E)-2-nonenal, 3-Nonen-2-one, (E)-2-octenal and Ethyl 3-hydroxybutanoate decreased or even disappeared to varying degrees on the fourth day. The decrease of aldehydes was more obvious and these substances belong to the main aroma of cucumber [50]. The results showed that the aroma of the samples was lost in different degrees during storage, which might be due to the destruction of cucumber tissue by microbial growth, thus leading to the decline of nutrients and aroma. Compared with the NaClO treatment and US-NaClO treatment, the content of aldehydes in the control group decreased rapidly, and the US-NaClO treatment group was better than the NaClO treatment group, indicating that US-NaClO treatment had a certain effect on maintaining the flavor of cucumber samples at the initial stage. When fresh-cut cucumber samples were stored in the late stage, ketones and alcohols, such as 3-pentanone, Isopropyl alcohol, n-hexanol, 1, 3-butanediol, 3-Octanone and other substances, would be produced. The increase of alcohols might be due to the aging of tissues. Some fatty acids are oxidized to alcohols by lipoxygenase, or the formation of alcohols by aldehyde under the action of deoxyenzymes. Compared with the control group, the content of ketones and alcohols in the NaClO treatment group and the US-NaClO treatment group was lower. In conclusion, the treatment group had a certain effect on maintaining the flavor of fresh-cut cucumber during storage, while the US-NaClO combined treatment was slightly better than the NaClO treatment group.

### 3.5. Sensory Analysis

**Figure 8:**
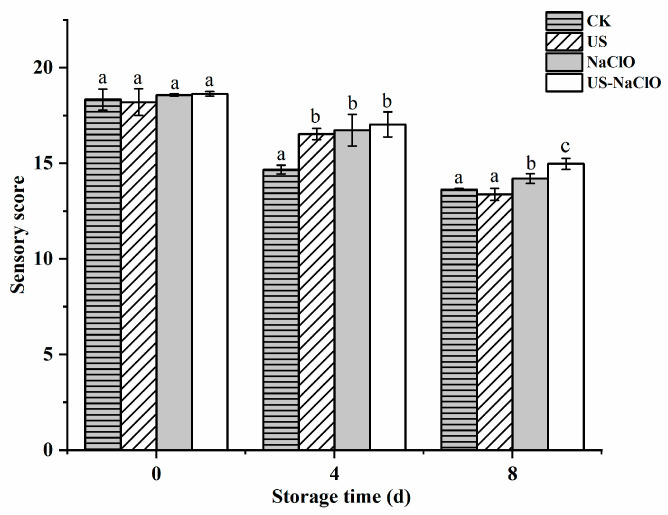
Effects of US, NaClO and US-NaClO treatment on sensory content of fresh-cut cucumber during storage. Different letters indicate significant differences among treatments at same storage time (*p* < 0.05).

The sensory characteristics (appearance, color, flavor, texture) of fruits and vegetables determine the shelf life of products and consumer acceptance, while fresh-cut vegetables are more prone to tissue softening, water loss, firmness decline and loss of fresh color and smell due to mechanical processing [32]. Figure 8 shows the sensory scores of fresh-cut cucumber samples under different treatments. The sensory scores of the control group, US, NaClO and US-NaClO groups had no significant difference on Day 0 (*p* > 0.05). At the end of the storage period, the sensory scores of the control group, US, NaClO and US-NaClO were 13.63, 13.37, 14.2 and 14.97, respectively. US-NaClO treatment was significantly better than other treatment groups. In general, US-NaClO treatment could reduce the water loss of cucumber to maintain its firmness, killed the odor generated by microbial degradation and effectively maintained the quality of fresh-cut cucumber during storage [51].

## 4. Conclusions

In conclusion, the concentration of NaClO was reduced by US binding sodium hypochlorite treatment in this study. US treatment for 5 min combined with 75 ppm NaClO compared with single treatment could effectively reduce the microbial growth of fresh-cut cucumber during storage and has no adverse effect on the quality. It maintains the integrity of the cell membrane by slowing down the content of malondialdehyde and water migration, reduces the increase of the weight loss rate to reduce water loss and maintains the firmness of fresh-cut cucumber during storage. Reducing the decrease of chlorophyll content had a positive effect on the color of fresh-cut cucumber. Similarly, it could effectively maintain the flavor of fresh-cut cucumber during storage. US-NaClO could inhibit the decline of aldehyde content of the cucumber’s main aroma substances and reduce the content of alcohols and ketones during storage to reduce the generation of bad odor. Therefore, US-NaClO treatment could provide theoretical and technical support for fresh-cut cucumber preservation and reduce production costs.

## Figures and Tables

**Table 1:** Weight loss and firmness of fresh-cut cucumber treated with US, NaClO, US-NaClO compared with the control during storage.

Storage Time (d)	Treatment	Weight Loss (%)	Firmness (N)
0	CK	0	4.88 ± 0.36 ^a^
	US	0	4.87 ± 0.34 ^a^
	NaClO	0	4.90 ± 0.43 ^a^
	US-NaClO	0	4.78 ± 0.23 ^a^
4	CK	3.02 ± 0.37 ^a^	4.09 ± 0.18 ^a^
	US	3.03 ± 0.52 ^a^	4.25 ± 0.29 ^ab^
	NaClO	2.67 ± 0.19 ^ab^	4.33 ± 0.35 ^ab^
	US-NaClO	2.04 ± 0.14 ^b^	4.43 ± 0.23 ^b^
8	CK	4.57 ± 0.19 ^a^	3.85 ± 0.26 ^a^
	US	4.65 ± 0.59 ^a^	4.20 ± 0.24 ^b^
	NaClO	4.09 ± 0.45 ^a^	4.29 ± 0.37 ^b^
	US-NaClO	3.21 ± 0.26 ^b^	4.34 ± 0.21 ^b^

Different superscripts in the same column indicate significant differences.

**Table 2:** Transverse relaxation time (T_23_) and free water proportion (A_23_) of fresh-cut cucumber during storage.

	Storage Time (d)	CK	US	NaClO	US-NaClO
T_23_ (ms)	0	1072.27 ± 0.00 ^a^	908.31 ± 54.32 ^b^	1125.79 ± 92.71 ^a^	1112.41 ± 80.29 ^a^
	4	1002.44 ± 98.76 ^b^	1125.79 ± 92.71 ^ab^	1232.85 ± 0.00 ^a^	1232.85 ± 0.00 ^a^
	8	871.87 ± 85.89 ^a^	851.62 ± 70.13 ^a^	871.87 ± 85.89 ^a^	758.31 ± 74.71 ^a^
A_23_ (%)	0	92.87 ± 0.26 ^b^	94.23 ± 0.33 ^a^	94.22 ± 0.70 ^a^	93.81 ± 0.30 ^a^
	4	94.88 ± 0.17 ^a^	94.91 ± 0.15 ^a^	93.60 ± 1.23 ^a^	89.05 ± 0.81 ^b^
	8	95.85 ± 0.46 ^a^	94.95 ± 0.33 ^a^	95.39 ± 0.35 ^a^	93.37 ± 0.79 ^b^

Different superscripts in the same column indicate significant differences.

**Table 3:** Qualitative information of volatile organic compounds in fresh-cut cucumbers under different treatments.

Count	Compound	CAS	Formula	MW	RI	RT/s	DT/ms
1	Hexanal	C66251	C_6_H_12_O	100.2	791.7	252.978	1.55912
2	Hexanal	C66251	C_6_H_12_O	100.2	800.9	260.662	1.25972
3	trans-2-pentenal	C1576870	C_5_H_8_O	84.1	746.7	218.579	1.35592
4	trans-2-pentenal	C1576870	C_5_H_8_O	84.1	750.2	221.082	1.10492
5	3-Pentanone	C96220	C_5_H_10_O	86.1	686.5	180.136	1.35242
6	3-Pentanone	C96220	C_5_H_10_O	86.1	690	181.898	1.11129
7	3-Methylbutanal	C590863	C_5_H_10_O	86.1	640.4	160.822	1.39938
8	3-Methylbutanal	C590863	C_5_H_10_O	86.1	641.3	161.168	1.19132
9	Isopropyl alcohol	C67630	C_3_H_8_O	60.1	500.7	114.007	1.23512
10	Isopropyl alcohol	C67630	C_3_H_8_O	60.1	497.6	113.143	1.08602
11	Heptanal	C111717	C_7_H_14_O	114.2	899.3	359.656	1.68588
12	Heptanal	C111717	C_7_H_14_O	114.2	901.2	362.014	1.33124
13	2-hexen-1-ol	C2305217	C_6_H_12_O	100.2	860.5	316.271	1.51138
14	2-hexen-1-ol	C2305217	C_6_H_12_O	100.2	865.9	321.93	1.17926
15	(E)-2-Heptenal	C18829555	C_7_H_12_O	112.2	957.7	442.956	1.65787
16	(E)-2-Heptenal	C18829555	C_7_H_12_O	112.2	958.1	443.574	1.25437
17	2-pentyl furan	C3777693	C_9_H_14_O	138.2	993.1	502.694	1.25191
18	2,4-Heptadienal	C5910850	C_7_H_10_O	110.2	1004.7	524.576	1.19846
19	(E,E)-2,4-heptadienal	C4313035	C_7_H_10_O	110.2	1018.2	551.77	1.19481
20	(E,E)-2,4-heptadienal	C4313035	C_7_H_10_O	110.2	1018.4	552.195	1.61153
21	2,4-Heptadienal	C5910850	C_7_H_10_O	110.2	1004.2	523.727	1.61639
22	3-Octanone	C106683	C_8_H_16_O	128.2	988.9	495.258	1.30659
23	3-Octanone	C106683	C_8_H_16_O	128.2	989.9	496.958	1.71237
24	(Z)-6-nonenal	C2277192	C_9_H_16_O	140.2	1103.6	758.983	1.16983
25	(Z)-6-nonenal	C2277192	C_9_H_16_O	140.2	1101.9	754.022	1.76907
26	Nonanal	C124196	C_9_H_18_O	142.2	1105.6	764.653	1.47671
27	Nonanal	C124196	C_9_H_18_O	142.2	1104.9	762.527	1.93431
28	(E,Z)-2,6-nonadienal	C557482	C_9_H_14_O	138.2	1144	882.299	1.37321
29	(E,Z)-2,6-nonadienal	C557482	C_9_H_14_O	138.2	1144.3	883.297	1.88471
30	octanal	C124130	C_8_H_16_O	128.2	1009.1	533.24	1.80799
31	octanal	C124130	C_8_H_16_O	128.2	1008.5	532.022	1.40866
32	Hexyl acetate	C142927	C_8_H_16_O_2_	144.2	1023.5	562.699	1.3826
33	4,5-dihydro-3(2H)-thiophenone	C1003049	C_4_H_6_OS	102.2	946.5	425.656	1.21219
34	n-hexanol	C111273	C_6_H_14_O	102.2	879.1	336.019	1.98374
35	Hexanenitrile	C628739	C_6_H_11_N	97.2	875.6	332.251	1.57373
36	n-hexanol	C111273	C_6_H_14_O	102.2	882.6	339.859	1.63788
37	1,3-butanediol	C107880	C_4_H_10_O_2_	90.1	788.7	250.523	1.36979
38	Butyl formate	C592847	C_5_H_10_O_2_	102.1	720.4	200.71	1.22857
39	2-methyl-1-Pentanol	C105306	C_6_H_14_O	102.2	827.3	283.957	1.59164
40	pentanal	C110623	C_5_H_10_O	86.1	703.3	189.923	1.40474
41	ethyl propanoate	C105373	C_5_H_10_O_2_	102.1	704.2	190.471	1.47653
42	2-methylbutanol	C137326	C_5_H_12_O	88.1	739.5	213.543	1.46241
43	isoamyl acetate	C123922	C_7_H_14_O_2_	130.2	875.8	332.381	1.7438
44	2,6-Dimethylpyrazine	C108509	C_6_H_8_N_2_	108.1	915.6	381.223	1.54727
45	(E)-2-nonenal	C18829566	C_9_H_16_O	140.2	1150.1	902.785	1.41067
46	3-Nonen-2-one	C14309570	C_9_H_16_O	140.2	1147	892.481	1.92945
47	(E)-2-nonenal	C18829566	C_9_H_16_O	140.2	1149.1	899.519	1.97249
48	(E)-2-octenal	C2548870	C_8_H_14_O	126.2	1064.6	656.046	1.33373
49	2-nonanone	C821556	C_9_H_18_O	142.2	1092.7	728.544	1.40615
50	Ethyl 3-hydroxybutanoate	C5405414	C_6_H_12_O_3_	132.2	943.4	420.887	1.17274
51	2-Methyl-2-propanol	C75650	C_4_H_10_O	74.1	521.7	120.069	1.12829

## Data Availability

Data is contained within the article.
